# Health state perception of people close to retirement age: Relationship with lifestyle habits and subjects’ characteristics

**DOI:** 10.1016/j.heliyon.2023.e17995

**Published:** 2023-07-17

**Authors:** Diana Monge-Martín, Fernando Caballero-Martínez, Maria João Forjaz, Manuel J. Castillo, Carmen Rodríguez-Blázquez

**Affiliations:** a Faculty of Medicine, Francisco de Vitoria University Foundation, Madrid, Spain; b Instituto de Salud Carlos III, National Center of Epidemiology, Madrid, Spain; c REDISSEC, Spain; d Faculty of Medicine, Universidad de Granada, Granada, Spain; e CIBERNED, Spain

**Keywords:** Ageing people, Lifestyle habits, Retirement, Perceived health, Healthy ageing

## Abstract

**Aim:**

Societal ageing increases the need for correct and healthy ageing to ensure the well-being of older adults. Practical strategies are needed to acquire healthy habits for the ageing process. This study aims to analyse the lifestyle habits of subjects who are retired or close to retirement and identify factors that could influence their perceived health and that could be related to these habits.

**Methods:**

A Spanish observational, descriptive, cross-sectional study of subjects close to retirement-age. Socio-demographic, family, work, leisure, social, and clinical-psychological indicators were evaluated.

**Results:**

1,700 participants (581 employed; 714 retirees; 405 other-status) were included, average age 63 years, 52% women. Most reported a satisfactory social life (90%), were in live-in relationships (74%), non-smoking (80%), followed a Mediterranean diet (73%), and took medicines daily (70%). Perceived health (EQ-VAS) was 75.9/100, with low disability (12-WHODAS) (7.4/100) and moderate/severe depression. Women reported higher disability (p < 0.001) and depression (p < 0.001), a better social life, and healthier lifestyle, but lower physical/work activity. Retirees reported less depression, better social life, healthier lifestyle, higher physical/work activity, and better sleeping habits. The multivariate model showed a significant association of health-status with disability level, number of chronic diseases, sleep habits, exercise, diet, and alcohol consumption. When depression level was introduced, age and being a woman were also related.

**Conclusions:**

Retirement does not mean worse health but rather an opportunity to reinforce favourable health activities and improve lifestyle factors. Incorporating the differences related to gender and employment status in health-perception will facilitate the design of healthy ageing strategies.

## 1 Introduction

The ageing of today's society, resulting from a low fertility rate and continued fall in the adult mortality rate, is unprecedented in human history [1]. On a global scale, the population growth rate of people over 60 years is twice that of the general population [2]. In the case of Spain, more than one-fifth of the population is over 60 years, with a life expectancy exceeding 80 years [3]. Moreover, 9.5 million people are over 65 years old, with a ratio of women to men of 57%:43%. In addition, despite the legal age of retirement in Spain being 65 years, the real age is closer to 63 years [3]. Adapting to this new demographic reality requires substantial changes in political, economic, social, and health paradigms [4].

Ageing is associated with a decrease in physical and mental capacity, as well as an increased risk of chronic-degenerative diseases and comorbidities [5]. However, ageing also represents a success of human progress, provided that it is accompanied by actions promoting healthy and active ageing that fosters functional capacity, social contribution, and quality of life in old age [5]. For these reasons, adopting healthy living habits during adulthood and old age can be a protective factor against disease (physical and mental), disability, and premature death [6]. In addition, adopting health-related factors impacts subjective well-being considerably [7].

The transition to retirement can be a challenging time involving anxiety, concerns, and social isolation. However, it may also offer the opportunity for a new, active, and positive phase of life, adopting healthier routines and habits to achieve successful aging [8]. Alternatively, it may also give rise to the maintenance or intensification of established unhealthy behaviours [9]. It is important to note that this transition is not the same for all retirees; there are intrinsic characteristics (health-related behaviours, traits and skills, physiological changes and risk factors, diseases, ethnicity, educational attainment, gender and wealth, etc.) [10] that differ among older adults and that could have more impact than chronological age per se [11]. In addition, the psychological and emotional state of the individual is very important during the last stage of life. In this sense, life-changing events such as inactivity due to retirement, an increase in chronic diseases, assumption of new family and social roles (being grandparents), loss of loved ones, or the feeling of being close to death can influence a person's psychological state and require both physical and emotional adjustment [12]. Older adults are considered to have better emotional control and psychological maturity, and the acquisition of healthy habits, such as the continued practice of certain abilities, can positively influence this process of growing maturity [12]. In Spain, more research is needed on the relationships among retirement, life habits, physical, and psychological health to design programs and strategies for the promotion of healthy lifestyles, with the resulting individual and social benefits they suppose. Based on our clinical experience with the retiree population, our hypothesis is that retirees are more predisposed to acquire healthy habits because they are more aware of the stage of life in which they are and therefore reinforce favourable health actions and correct unhealthy behaviours.

For this reason, this study was carried out to identify lifestyle habits that may impact the self-perceived health status (clinical and psychological) in a population with an age close to retirement or already retired.

## 2 Materials and methods

### 2.1 Study design and population

A descriptive, analytical, national, cross-sectional survey was carried out following approval by the Research Ethics Committee of the Universidad Francisco de Vitoria (Madrid) (ethical approval number: 13/2019).

The sample was obtained by proportional multistage stratified sampling based on geographical location. The sample size (1,959 individuals) was randomly selected based on eligibility criteria from a target population of 10,506,015 people aged between 55 and 75 years ( ± 10-years relative to the average retirement age in Spain) in 2020. Data collection was carried out between July-2019 and February-2020. The eligibility criteria were subjects of both sexes, between 55 and 75 years old. These were also the recruitment criteria provided to the digital market research platform. Individuals meeting these criteria were candidates to receive an online survey designed by the researchers and conducted by an international consumer digital market research platform. An email was sent containing a study survey link. Those interested filled out the survey. Eligible subjects were contacted by platform staff by phone and were informed of the study purposes. Eligible participants were required to state their verbal informed consent. All participants needed access to the Internet and had to be familiar with its use.

The data were collected through a structured online 55-item survey, including socio-demographic, family, work, leisure, social participation, lifestyle, and health indicators questions.

Socio-demographic data included: gender, age, marital status (married, single/separate/divorced, widowed), educational level (primary or below, secondary, university), and employment status (active workers, unemployed, retiree, family and home care, pensioner). A retiree is a person who terminates employment and has retired from a working or professional career. In Spain, the legal retirement age is 65 years, but in some cases, people retire between two to three years before this. The State provides the pension. A pensioner is a working person who for health reasons stops working before the retirement age after undergoing a specific medical assessment that. The pensioner is assessed as not fit to work and receives a lower allowance from the State for the rest of his/her life. Social and leisure characteristics (social activity and sexual life) were also collected.

Information on comorbidities, number of medications per day, and self-medication were requested to assess physical health. The 12-item Self-Report World Health Organization Disability Assessment Schedule (12-WHODAS) was used for disability degree determination [13], scoring from 0 to 100 (0 = no disability, 100 = total disability). For mental health assessment, the 0 to 27 point scale, Patient Health Questionnaire [PHQ]-9, was used (0 = no depression, 1–4 = minimal, 5–9 = mild, 10–14 = moderate, 15–19 = moderate/severe, and 20–27 = severe depression]) [14].

Lifestyle-related variables included sleep, physical activity, tobacco, alcohol consumption (yes/no) in the last 12 months, and eating habits.

Self-perceived health by the participants was determined using the EuroQol-visual analogue scale (EQ-VAS) of the EQ-5D-5L questionnaire, with scores ranging from 0 (worst imaginable health state) to 100 (best imaginable health state) [15].

### 2.2 Statistical analysis

Variables were described using summary statistics such as counts, mean, and standard deviation (SD) for continuous variables, and counts and percentages for categorical variables.

A general description of the study sample was made, followed by an analysis of the sample stratified by gender (male, female) and by employment status (workers and retirees). In these subgroups, socio-demographic, family, educational, social and leisure variables, and health indicators were compared using the chi-squared test for categorical variables. Moreover, analysis of variance (ANOVA) was performed for quantitative variables (age, number of chronic pathologies, PHQ-9, or self-perceived health state [EQ-VAS]). Non-parametric equivalent tests used were Kruskal Wallis and Mann-Whitney U. Bonferroni correction was used for multiple peer comparisons.

Two multivariate linear regression models were performed for the total sample with self-perceived health state (EQ-VAS) as a dependent variable: one including socio-demographic variables, disability level, comorbidities, lifestyle habits, and health indicators as independent variables (model 1), and a second adding depression level, assessed by PHQ-9 scale (model 2).

An additional independent regression model was created considering only the group of workers and retirees (excluding unemployed, family and home care, and pensioners). Stratified models for each subgroup were also performed. Tests were conducted to ensure that the basic assumptions of linearity, normality, and independence of the multiple linear regression model were met. To facilitate the interpretation of the models, the standardised β coefficients were displayed for each variable.

The minimum level of significance was set at p < 0.05, and version 20 of the IBM SPSS for Windows statistical package was used.

## 3 Results

Of 1,959 potential participants, 1,700 subjects completed the survey.

### 3.1 Socio-demographic characteristics

Table 1 reflects the sample's socio-demographic characteristics and lifestyle habits and is stratified by gender and employment status.

**Table 1:** Socio-demographic characteristics and lifestyle habits of the sample (overall and stratified by gender and working status) and Health state (general [EQ-VAS scale], clinical [12-WHODAS scale], and psychological [PHQ-9 questionnaire]) perceived by participants, overall and based on employment status.

	N = 1700				N = 1295		
	General	Male N = 816	Female N = 884	p**	*Active Workers* *N=581*	*Retirees* *N=714*	P**
**Gender, n (%)**							0.818
**Male**	816 (48)	NA	NA		312 (53.7)	388 (54.3)	–
**Female**	884 (52)	NA	NA		269 (46.3)	326 (45.7)	–
**Age (years), mean ± SD**	63.3 ± 5.7	63.4 ± 5.7	63.1 ± 5.7	0.228	58.7 ± 3.5	67.5 ± 3.9	<0.001
**Marital status, n (%)**				<0.001			
Married - in a couple	1,265 (74.4)	672 (82.4)	592 (67)		450 (77.5)	525 (73.5)	0.003
Single/separate/divorced	325 (19.1)	119 (14.6)	206 (23.3)		–	–	–
Widowed	110 (6.5)	25 (3.1)	86 (9.7)		–	51 (7.2)	–
**Educational level, n (%)**				<0.001			
Primary or below	126 (7.4)	42 (5.2)	83 (9.4)		16 (2.7)	68 (9.5)	NS
Secondary	860 (50.6)	396 (48.5)	464 (52.5)		278 (47.9)	345 (48.3)	NS
University	714 (42)	378 (46.4)	337 (38.1)		287 (49.4)	301 (42.2)	<0.001
**Employment status, n (%)**				<0.001	NA	NA	NA
Active workers	581 (34.2)	312 (38.2)	269 (30.4)		NA	NA	NA
Unemployed	122 (7.2)	47 (5.7)	77 (8.7)		NA	NA	NA
Retiree	714 (42.0)	388 (47.6)	326 (36.9)		NA	NA	NA
Family and home care	129 (7.6)	6 (0.7)	123 (13.9)		NA	NA	NA
Pensioner	153 (9.0)	63 (7.8)	89 (10.1)		NA	NA	NA
**Chronic diseases**							
Nº (average) per individual (SD)	3.5 ± 2.5	3.2 ± 2.2	3.8 ± 2.7	<0.001	3.1 ± 2.4	3.7 ± 2.5	<0.001
Bone, n (%)	728 (42.8)	235 (28.8)	493 (55.8)	<0.001	200 (34.4)	323 (45.2)	<0.001
HBP, n (%)	619 (36.4)	353 (43.3)	265 (30)	<0.001	176 (30.3)	306 (42.8)	0.002
High cholesterol, n (%)	583 (34.3)	260 (31.9)	325 (36.5)	0.042	198 (34.1)	245 (34.3)	0.929
Insomnia, n (%)	437 (25.7)	147 (18)	289 (32.7)	<0.001	137 (23.6)	179 (25.1)	0.535
Vision problems, n (%)	369 (21.7)	150 (18.4)	219 (24.8)	0.001	98 (16.8)	174 (24.4)	0.001
**Daily medication consumption, n (%)**	1136 (66.8)				350 (60.2)	520 (72.8)	<0.001
1-2 medications daily	884 (52.0)	386 (47.3)	536 (57.4)	0.001	374 (64.4)	324 (45.4)	NS
≥3 medications daily	816 (48.0)	430 (52.7)	348 (42.6)		207 (35.6)	390 (54.6)	
**Sleep problems in the last month, n (%)**	224 (13.2)	–	–	–	70 (12.1)	84 (11.8)	–
**Physical activity, n (%)**				0.02			
Exercises ≥3 days/week	889 (52.3)	449 (55.0)	442 (50.0)		259 (44.5)	408 (57.2)	<0.001
Exercises ≤2 day/week,	459 (27.0)	220 (27.0)	239 (27.0)		185 (31.9)	178 (25.0)	–
Does not exercise (sedentary)	352 (20.7)	147 (18.0)	203 (23.0)		137 (23.6)	127 (17.8)	–
**Eating habits, n (%)**				0.003			
Very good diet (Mediterranean diet)	1246 (73.3)	571 (70.0)	672 (76.0)	–	397 (68.3)	547 (76.6)	0.004
Diet could be improved	442 (26.0)	245 (30.0)	203 (23.0)	–	178 (30.6)	161 (22.6)	–
Unhealthy diet	17 (1.0)	8 (1.0)	9 (1.0)	–	6 (1.0)	57 (0.8)	–
Alcohol consumption* (last year), n (%)	1331 (78.3)	690 (84.6)	641 (72.5)	<0.001	485 (83.4)	562 (78.7)	<0.001
**Tobacco consumption, n (%)**				<0.001			
Not daily	27 (1.6)	10 (1.2)	17 (1.9)		20 (3.4)	4 (0.5)	NS
Daily	333 (19.6)	164 (20.1)	169 (19.1)		148 (25.5)	98 (13.7)	<0.001
Ex-smoker	785 (46.2)	458 (56.1)	329 (37.2)		253 (43.5)	373 (52.3)	NS
Non-smoker	559 (32.6)	184 (22.6)	369 (41.8)		161 (27.7)	239 (33.5)	NS
**Social life, n (%)**				0.024			–
Satisfactory (stays active)	884 (52.0)	416 (51.0)	477 (54.0)		291 (50.1)	408 (57.1)	0.049
Quiet (prefers to spend time alone)	663 (39.0)	343 (42.0)	318 (36.0)		233 (40.1)	253 (35.4)	–
Hardly any social life, although he/she would like one	136 (8.0)	49 (6.0)	80 (9.0)		48 (8.3)	48 (6.7)	–
Bad (lonely, with no one to turn to)	17 (1.0)	8 (1.0)	9 (1.0)		9 (1.5)	57 (0.8)	0.049
**Sex life, n (%)**				<0.001			
Very good/Good	775 (45.6)	400 (49)	371 (42)		303 (52.2)	287 (40.2)	<0.001
Normal	527 (31)	269 (33)	256 (29)		173 (29.8)	237 (33.2)	–
Bad/Very bad	340 (20)	147 (18)	247 (28)		102 (17.6)	190 (26.6)	–
**EQ-VAS, mean (SD)**							
**- Overall**	75.9 ± 18.3	76.5 ± 17.2	75.4 ± 19.3	0.484	77.2 ± 17.0	75.5 ± 18.7	0.389
**Based on employment status,**	<0.0001				NA	NA	NA
Active	77.2 ± 17.0	77.1 ± 15.5	77.3 ± 18.5		NA	NA	NA
Unemployed	79.6 ± 16.8	79.1 ± 17.4	79.9 ± 16.5		NA	NA	NA
Retired	75.5 ± 18.7	76.3 ± 17.7	74.6 ± 19.8		NA	NA	NA
Family and home care	73.5 ± 17.7	73.2 ± 20.2	73.5 ± 17.7		NA	NA	NA
Pensioner	71.5 ± 21.9	72.6 ± 20.9	70.8 ± 22.7		NA	NA	NA
**WHODAS, mean ± SD**	7.4 ± 11.4	6.5	8.2	<0.001^a^	6.89 ± 11.4	7.3 ± 11.5	0.662^a^
**PHQ-9, n (%)**							
No	527 (31)	302 (37)	221 (25)	<0.001^a^	154 (26.5)	261 (36.5)	0.001^a^
Minimal	799 (47)	375 (46)	415 (47)		282 (48.6)	325 (45.5)	
Mild	272 (16)	106 (13)	159 (18)		103 (17.7)	95 (13.3)	
Moderate	85 (5)	24 (3)	53 (6)		32 (5.6)	21 (2.9)	
Moderate/Severe	17 (1)	8 (1)	18 (2)		6 (1.1)	9 (1.2)	
Severe	17 (1)	0 (0)	9 (1)		2 (0.4)	6 (0.8)	

**SD**: Standard deviation; **HBP**: high blood pressure; **NS**: not significant; **EQ-VAS**: visual analogue scale to assess quality of life; **12-WHODAS**: 12-item Self-Report World Health Organization Disability Assessment Schedule; **PHQ-9**: Patient Health Questionnaire, 9 items.

*Consumption of any kind of alcoholic drink, even if only in exceptional circumstances.

**t-student, Mann-Whitney U and Chi-squared tests.

a Mann-Whitney U, Kruskal Wallis and Chi-squared tests.

The sample included a similar number of women and men (n = 884, 52%/n = 816, 48%). The mean age was 63 years (SD = 5.7), which was very similar between genders; however, the active workers subgroup was approximately 10 years younger than retirees (Table 1). Employment statuses were ‘other’ in 405 subjects. Among the 1,295 subjects with this data, 443 (34.2%) were workers, and 544 (42%) were retired (highest percentage in men; p < 0.001). However, more women were unemployed, pensioners, and family/home carers (p < 0.001) (Table 1).

Overall, 1,265 (74.4%) subjects were married/partnered, with the highest rates in men (82.4%) and workers (77.5%) (Table 1). The percentage of subjects living alone was higher in retirees vs. workers (17.6% vs. 8.8; p < 0.001).

Overall, the educational level was mainly secondary in 903 (51%) subjects or university in 714 (42%) subjects; with a significantly higher percentage of university studies in men and workers (Table 1). In contrast, the percentage of women with primary education was significantly higher (9.4% vs. 5.2%) (Table 1).

### 3.2 Health status

The mean of chronic diseases was 3.5 (higher in women and retirees; p < 0.001). The most common comorbidities were bone pain and high blood pressure (Table 1). Medication was taken daily by 1,136 (66.8%) participants (>50% 1–2 medications, most commonly by women; p < 0.001) (Table 1). Retirees most commonly took ≥5 medications daily versus workers (19% vs. 9.2%; p < 0.001). Self-medication was reported by 952 (56%) subjects.

Data on workers' and retirees' self-perceived clinical and psychological health levels are presented in Table 1. Overall, the mean score of the self-perceived health state (EQ-VAS) was 75.9, without statistically significant differences between genders. The worst and the best status were stated by pensioners and unemployed, respectively (p < 0.001). No differences were detected in the sample stratified in workers and retirees (Table 1).

Regarding disability degree (12-WHODAS scale), the mean score was 7.4 (SD = 11.4). Women reported a higher value than men (8.2 vs. 6.5, p < 0,001), without statistically significant differences between workers and retirees (Table 1).

The PHQ-9 questionnaire showed that 7% of participants might be diagnosed with moderate to severe depression, most common in women (p < 0.001) and workers (p = 0.001) (Table 1).

### 3.3 Lifestyle

There were differences in certain health habits, such as tobacco (daily) and alcohol (last year) consumption, which were lower in women (p < 0.001) and retirees (p=<0.001) (Table 1). The percentage of sedentary lifestyles was higher in women (18% vs. 23%, p = 0.02) and retirees (17.8% vs. 23.6%, p < 0.001). Following a healthy diet was more common in women (p = 0.003) (Table 1) and retirees (p = 0.004) (Table 1).

### 3.4 Social and leisure characteristics

There were differences in the perception of sex life, worse in women and retirees (p < 0.001); however, both stated better social life, keeping them active (p = 0.024 and 0.049, respectively).

### 3.5 Associations of perceived health status with socio-demographic and health variables-Linear regression models

Table 2 reflects the results of the linear regression models of self-perceived health status (EQ-VAS) (overall and according to employment status). A strong association was found between the subject's health status and disability level, number of chronic diseases, sleeping habits, exercise, diet, and alcohol consumption (Table 2. Model 1). When analysing the depression level (percentage of variance), the model increased from 33.3% (Model 1) to 36.3% (Model 2), with age and sex appearing as new variables associated with self-perceived health status (Table 2. Model 2).

**Table 2:** Linear regression model of self-perceived health status (EQ-VAS) by the respondents; overall (n = 1,700) and stratified by working status (subsample n = 1,295).

	Standardised β coefficients n = 1,700		Standardised β coefficients n = 1,295		
	Model 1^a^	Model 2^a^	*Model 2 subsample*^c^		
			*Total subsample*	*Workers n=581*	*Retirees n=714*
**Socio-demographic variables**					
Gender	−0.033	−0.049*	−0.054*	−0.098*	−0.023
Age	−0.033	−0.048*	−0.039	−0.009	−0.031
Educational level	0.007	0.006	0.017	0.031	−0.003
Marital status	−0.037	−0.033	−0.020	−0.035	−0.019
Employment status	0.000	0.003	0.018	–	–
**Health variables**					
Disability: 12-WHODAS	−0.313*	−0.243*	−0.238*	−0.191*	−0.259*
Nº Chronic diseases	−0.273*	−0.214*	−0.221*	−0.181*	−0.240*
Sleep	0.115*	0.066*	0.084*	0.030	0.116*
Smoking	−0.021	−0.022	−0.021	−0.053	0.006
Depression; PHQ-9	–	−0.212*	−0.204*	−0.307*	−0.149*
Exercises ≥3 days/week	0.092*	0.083*	0.070*	0.102*	0.045
Healthy diet	0.067*	0.043*	0.051*	0.001	0.088*
Alcohol (wine or beer with meals)	0.061*	0.056*	0.073*	0.038	0.095*
Adjusted R^2^	0.333^b^	0.363^b^	0.348	0.339	0.359

*p < 0.05.

**12-WHODAS** = 12-item Self-Report World Health Organization Disability Assessment Schedule; **EQ-VAS** = visual analogue scale to assess quality of life; **PHQ-9:** Patient Health Questionnaire, 9 items.

a Linear regression model taking into account the level of depression (**model 2**) or not (**model 1**) according to the PHQ9 questionnaire.

b F change = 65,857, p < 0.001.

c Linear regression model stratified by working status (subsample of workers and retirees).

The best perceived health status in workers (explained variance = 33.9%) and retirees (explained variance = 35.9%) subgroups was associated with having a lower disability and depression degree and a lower number of chronic diseases (Table 2). Moreover, in the workers subgroup, being a woman and exercising ≥3 times/week were also associated with this. In the retiree subgroup, a healthy diet (Mediterranean diet), drinking some wine or beer at mealtimes, and sleeping well were associated with the best perceived health status.

## 4 Discussion

The present study provides socio-demographic and clinical profile data of a countrywide sample of the adult and older adult population with an age close to retirement or recently retired. It also provides information on factors that influence self-perceived health status.

Of 1,700 respondents, the proportion of women and men was similar, despite women being the majority of older adults [16]. As previously reported in the older adult population [17], women showed lower educational attainment and were more likely to be engaged in domestic work, unemployed, or pensioners than men. These results are consistent with Spanish and European official data (EU-28) [3], reflecting the sample representativeness. The higher representation of women in domestic work, unemployment, or pensioners may explain the higher percentage of working or retired men (although this was not significant).

The self-perceived health status in our population was higher than previously reported in Spain [18]. This may be due, in part, that those data [18] came from a study published in 2009 [19], when the economic and social situation in Spain was worse due to the global financial crisis of 2008 - a circumstance that could interfere with the self-perceived health status at that time.

Overall, the best perceived health status was associated with lower disability, fewer chronic diseases, sleeping well, increased weekly exercise, and healthy eating with moderate wine or beer consumption during meals. Similar associations have been found, where perceived health was strongly associated with physical and functional health, among others [20].

Comparing perceived health status between genders showed a slightly non-significant higher value in men, consistent with previous studies showing a worse self-perception of health in women [18]. This could be explained, among other reasons, by women having a greater care burden and lower education level. The latter has been reported to be related to a worse psychological adjustment concerning well-being in older people [21]. Although a reported cause of this worse perception of health in Spanish women is having less leisure time [22] in our sample, women had a significantly slightly better social life, in line with the previous increase in their social life reported (without statistical significance) [1]. This more positive leisure attitude, which influences the psychological well-being of older people [21], may contribute to the difference in the perceived health status between genders not being significant. The observed healthier diet in women, in line with the higher consumption of vegetables and fish described in women and higher consumption of processed and red meat in men [23], can also influence this. These findings suggest a need to design health promotion programs selectively aimed at improving men's eating habits, particularly with training in basic culinary skills for those who live alone, as it is suggested that men without partner are more likely to acquire risk behaviours [24].

Another possible cause for the better self-perceived health state of men could be greater physical activity [25]. In our population, men were slightly more active than women. Moreover, the absence in men of menopause-associated problems (osteoporosis and neuro-vegetative symptoms) [26] may also impact this better perception. These menopause-associated disorders, along with other frequent age-specific comorbidities (including gynaecological cancers) affecting sexual satisfaction [26], may also be a reason why women reported a worse sexual life.

With ageing, biological changes in the human body affect mood, physical condition, and social activity that influence one's perception of health [27]. In Spain, depression takes ninth place in chronic diseases and is more prevalent in women [3]. This phenomenon is reflected in our study: although the percentage of moderate to severe depression is low (<1 in 10 participants), it was higher in women. In this sense, women are more likely to suffer from non-lethal disabling conditions such as depression [20]. When depression was included in our analysis, the model showed that a better perception of one's health status was associated with less depression, being a woman, and being younger. This is consistent with what has been published by other authors who have mentioned that women who do not suffer depression have a better health status perception [20].

Regarding the self-perceived health status according to employment status, the best health status in workers and retirees was associated with a lower disability degree, fewer chronic diseases, and lower depression levels. In workers, this perception was also related to being a woman and exercising ≥3 times/week. In retirees, it was related to a healthy diet, adequate sleep, and alcohol consumption.

Overall, retirees reported better self-perceived health than workers, with lower depression and disability levels, although in both groups the average disability level was very low.

The better perceived health reported by the retirees vs. workers may be explained by their healthy lifestyle (more physical activity, healthier eating habits, less tobacco and alcohol consumption). The many positive effects of habitual moderate exercise are well known [28]. In addition, a healthy diet, such as the Mediterranean diet, has protective effects in older adults, independent of whether they have a chronic disease [29]. These habits could counterbalance the deterioration that ageing supposes, as reflected in the significantly higher average number of chronic diseases stated by retirees (osteoarticular, vision, and hypertension). This is expected, given the association of ageing with gradual body deterioration, changes in organs and systems [30], and a greater burden of chronic diseases [31], including auditive/vision problems [32].

In workers and retirees, a better health status was associated with a lower degree of depression, which was significantly lower in those who were retired. However, there are conflicting data on the prevalence of depression in retirement. Despite being related to the loss of employment and the social life associated with work, retirement is also seen as an escape from work (source of daily stress, obligations, and responsibilities) [33]. In our case, the most plausible explanation is the freedom from work, with a significantly more satisfactory social life and exercised more.

A limitation of the study is that the sample analysed comprises people who volunteered to participate in computer surveys. This may have resulted in the over-representation of a population with a higher educational level, as they require access to and familiarity with the use of the Internet and new technologies (bias in the overall population representativeness), which may, in some way, impact the external validity of the results. Other limitations are the cross-sectional study design and not being able to conduct a causality analysis; a comparison stratified by rural vs. urban area has not been carried out; the alcohol consumption has not been measured quantitatively (only yes/no in the last year); and the mental health analysed is self-reported and therefore not quantifiable with cognitive scales. Despite these limitations, the study has many strengths and presents an update of the self-perceived health status in the Spanish population of those close to retirement or recently retired, identifying areas in which to take action to correct certain problems or unmet needs associated with this population.

## 5 Conclusions

The results of this study provide new and valuable information on the determinants of self-perceived health in the adult and mature stages of life.

Overall, the best perceived health status is associated with a lower degree of disability and depression, fewer chronic diseases, exercising ≥3 times/week, a healthy diet, and moderate alcohol consumption. A lower level of depression, being a woman, and being younger were also associated with a better perception of health status.

The perception of clinical health status was similar among genders; however, women reported less disability and more depression. Women reported doing less physical exercise, having a better lifestyle (healthier diet and less tobacco and alcohol consumption) and social life, but a worse sex life.

Our result showed that retirement does not necessarily have a detrimental effect on health status. Overall, retirees showed better overall health and lower depression than those working, moreover, they reported less depression, a healthier lifestyle (increased physical activity, healthier diet, less tobacco, and alcohol consumption), and better perception of their social life. Our results clearly reflect how retirement can be a vital opportunity to reinforce favourable health actions and correct unhealthy behaviours.

It is important to act upon all identified factors to help improve health status and achieve satisfactory ageing. To achieve this, specific strategies for each group are recommended, encompassing preventive, therapeutic, and advisory approaches that would promote change in unhealthy lifestyles.

## Summary of what is in the paper

The results of this study provide valuable new information on the determinants of perceived health in the adult and mature stages of life. They clearly reflect how retirement can be a vital opportunity to reinforce health-promoting actions and correct unhealthy behaviours. It is important to act on all the factors identified to help improve health status and achieve successful ageing.

## Author contribution statement

Diana Monge: Conceived and designed the experiments; Performed the experiments; Analyzed and interpreted the data; Wrote the paper.

Fernando Caballero: Conceived and designed the experiments; Wrote the paper.

Maria João Forjaz, Carmen Rodriguez-Blázquez: Conceived and designed the experiments; Analyzed and interpreted the data; Contributed materials, analysis tools or data; Wrote the paper.

Manuel J. Castillo: Contributed materials, analysis tools or data; Wrote the paper.

## Data availability statement

The data that has been used is confidential.

## Declaration of competing interest

The authors have no interests to declare.
